# Epigenetic regulation in medulloblastoma: from tumor heterogeneity to diagnostic, prognostic, and translational applications

**DOI:** 10.3389/fnmol.2026.1909580

**Published:** 2026-08-19

**Authors:** Xinling Zhang, Lailing Du, Kewei Tian, Lening Chen, Xiaoping Li

**Affiliations:** Key Laboratory of Artificial Organs and Computational Medicine in Zhejiang Province, Shulan International Medical College, Zhejiang Shuren University, Hangzhou, China

**Keywords:** clinical translation, diagnostic biomarkers, epigenetic regulation, medulloblastoma, molecular subgroups, targeted therapy

## Abstract

Medulloblastoma (MB) is the most common malignant pediatric brain tumor. Epigenetic dysregulation, particularly in Group 3 and Group 4 tumors, is a major driver of tumorigenesis despite relatively few recurrent driver mutations. DNA methylation, histone modification, chromatin remodeling, and non-coding RNAs orchestrate aberrant transcriptional programs governing tumor initiation, progression, and cellular identity. Single-cell and multi-omic studies have revealed epigenetic heterogeneity, cellular plasticity, and microenvironmental interactions underlying therapeutic resistance. Epigenetic alterations provide valuable diagnostic and prognostic biomarkers. DNA methylation profiling is the gold standard for molecular classification, while epigenetic signatures and cerebrospinal fluid circulating tumor DNA support precision diagnosis and disease monitoring. This review integrates recent advances in MB epigenetics into a framework linking cellular plasticity and the tumor microenvironment to biomarker-driven precision therapies. Several comprehensive reviews have summarized the epigenetic landscape of medulloblastoma, focusing primarily on DNA methylation, histone modifications, chromatin remodeling, and subgroup-specific epigenetic alterations. While these studies have substantially advanced our understanding of epigenetic mechanisms, the rapid emergence of single-cell sequencing, spatial transcriptomics, multi-omic integration, and three-dimensional chromatin mapping has reshaped the current view of medulloblastoma biology. These technologies provide unprecedented resolution for dissecting intratumoral heterogeneity, developmental trajectories, and dynamic epigenetic regulation not fully addressed in earlier reviews. In this review, we integrate these recent advances into a unified framework connecting classical epigenetic mechanisms with emerging multidimensional epigenomic technologies. We discuss how these insights facilitate biomarker discovery, improve molecular classification, and accelerate precision epigenetic therapies, highlighting future directions for translational research.

## 1 Introduction

Medulloblastoma is the most common malignant tumor of the cerebellum in children. It has been better understood from a molecular point of view than from a histopathological classification. The present consensus divides medulloblastoma into four molecular subgroups: WNT, SHH, Group 3, and Group 4, each with distinct oncogenic drivers, developmental origins, and clinical outcomes (Juraschka and Taylor, 2019). This classification is based on integrated genomic, transcriptomic, and epigenetic profiling, which dramatically enhanced the diagnostic and risk stratification capabilities (Northcott et al., 2017). Indeed, compared to the WNT and SHH subgroups, Group 3 and Group 4 tumors harbor relatively few recurrent driver mutations, implicating epigenetic regulation as a major determinant of their tumorigenesis and progression (Hovestadt et al., 2020; Northcott et al., 2017). These data reveal that, in addition to genetic alterations, epigenetic programs significantly mold MB’s transcriptional and cellular states. Increasing evidence indicates that epigenetic mechanisms contribute to tumor heterogeneity, lineage specification, and adaptive plasticity during disease progression (Hovestadt et al., 2019; Vladoiu et al., 2019). Therefore, epigenetic dysregulation remodeling chromatin states and gene expression programs has emerged as an important determinant of MB pathogenesis.

Epigenetic dysregulation involves reversible molecular changes such as aberrant DNA methylation, histone modification imbalance, chromatin structure disorganization, and non-coding RNA expression changes. These mechanisms affect chromatin accessibility and gene expression without any changes in the underlying DNA sequence, thus modulating cellular identity and oncogenic phenotypes. Characteristic epigenetic changes in medulloblastoma include genome-wide hypomethylation of DNA and locus-specific hypermethylation of tumor suppressor genes (Schwalbe et al., 2017). In addition, reduced 5-hydroxymethylcytosine (5hmC) levels have been linked with increased proliferation and poor clinical outcomes, thus making it a candidate prognostic biomarker (Zhao et al., 2021). Other epigenetic mechanisms contributing to tumorigenesis include histone modifier dysregulation. Several PRC2 components and histone demethylases, such as UTX/KDM6A and JMJD3/KDM6B, are often dysregulated, connecting epigenetic regulation with aberrant transcriptional programs (Dubuc et al., 2013; Robinson et al., 2012). Such coordinated epigenetic changes induce tumor growth, suppress differentiation, and promote survival, thus maintaining malignant phenotypes. Because epigenetic changes are reversible, they are good therapy targets; thus, epigenetic-based therapeutics have gained more attention (Lin et al., 2016).

Single-cell sequencing technologies and multi-omics approaches have recently shed light on the epigenetic landscape of medulloblastoma with unprecedented resolution. These findings reveal the complex interplay among molecular subgroups, tumor heterogeneity, and clinical outcomes. Differential expression of epigenetic regulators was tightly associated with subgroup identity and prognosis; for example, proliferative, stem-like malignant cells in SHH tumors bear epigenetic signatures associated with poor clinical outcomes (Riemondy et al., 2022). Indeed, integrative analyses of transcriptomic and DNA methylation data suggest that Group 3 and Group 4 medulloblastomas exist in a transcriptional continuum that recapitulates the discrete stages of fetal cerebellar development (Aldinger et al., 2021; Jessa et al., 2019). Disruption of developmental epigenetic programs is central to dictating biological behavior (Smith et al., 2022; Williamson et al., 2022). Other recent studies have suggested that aberrant enhancer activity, dysregulated chromatin accessibility, and an altered landscape of histone modifications converge to maintain lineage-specific transcriptional states and block normal neuronal differentiation (Hovestadt et al., 2019; Vladoiu et al., 2019).

These epigenetic aberrations induce cellular plasticity and developmental arrest and hence contribute to intratumoral heterogeneity, tumor progression, and therapeutic resistance.

This extends beyond transcriptional regulation to higher-order chromatin organization. Recent studies indicate that alterations in 3D chromatin organization contribute to subgroup-specific regulatory programs in medulloblastoma. However, current evidence supports a mechanistic role in transcriptional regulation rather than its use as an established approach for molecular subgroup discrimination. This emphasizes the role of spatial genome organization as another layer of epigenetic regulation in medulloblastoma. Furthermore, recent data suggest that epigenetic regulation and metabolic reprogramming are interlinked. Metabolic intermediates act as essential cofactors for epigenetic enzymes, creating a feedback loop that reinforces aberrant epigenetic states, driving tumor growth (Bunt et al., 2012; Faubert et al., 2020).

Rapid advances in single-cell sequencing, spatial transcriptomics, and integrative multi-omic technologies have substantially expanded our understanding of the dynamic epigenetic landscape, cellular plasticity, developmental hierarchies, and tumor heterogeneity, yet these emerging findings have not been comprehensively synthesized within a translational framework. In this review, we integrate recent discoveries in single-cell and multi-omic profiling with advances in epigenetic therapeutics, immune regulation, and clinically relevant biomarkers. By connecting fundamental epigenetic mechanisms with tumor evolution, therapeutic resistance, and precision medicine, we provide an updated perspective that bridges mechanistic insights and translational applications in medulloblastoma.

## 2 Core epigenetic regulatory mechanisms of medulloblastoma

Epigenetic dysregulation is a key driver of MB beyond genetic alterations. DNA methylation, histone modifications, chromatin remodeling, and non-coding RNA regulation collectively shape transcriptional programs and cellular identity, disrupt development, and promote tumor growth and plasticity, providing a foundation for understanding MB biology and identifying therapeutic targets (Hovestadt et al., 2020; Northcott et al., 2019).

### 2.1 DNA methylation dysregulation

Epigenetic modification by DNA methylation is one of the significant epigenetic aberrations in MB, resulting in genomic instability and transcriptional deregulation. Genome-wide hypomethylation is often found, inducing chromosomal instability, increasing mutation rates, and aberrant activation of oncogenic pathways, including MYC (Cho et al., 2011; Northcott et al., 2017). Genome-wide hypomethylation is frequently observed in medulloblastoma, contributing to chromosomal instability, increased mutation rates, and transcriptional deregulation. It may also promote the aberrant activation of normally silenced repetitive elements, a mechanism widely recognized in human cancers, although direct evidence in medulloblastoma remains limited (Hovestadt et al., 2014).

On the other hand, locus-specific promoter hypermethylation commonly silences tumor suppressor genes such as Ras Association Domain Family Member 1 Isoform A (RASSF1A), Hypermethylated In Cancer 1 (HIC1), and p16 Inhibitor of CDK4 (p16INK4a), particularly in SHH and Group 3/4 subgroups (Frühwald et al., 2001; Lusher et al., 2002; Rood et al., 2002). This results in the suppression of transcription factor binding and impairs the normal pathways of cell cycle control, DNA repair, and apoptosis, allowing tumors to grow continuously.

DNA methylation profiling has been among the most robust and clinically applicable tools in MB. High-resolution methylation-based classification distinguishes WNT, SHH, Group 3, and Group 4 subgroups, often surpassing conventional histopathology (Capper et al., 2018). More and more, methylation signatures are proving to have prognostic value and are being incorporated into risk stratification and clinical decision-making, underscoring their pivotal role in precision neuro-oncology (Capper et al., 2018; Schwalbe et al., 2017).

### 2.2 Histone modifications and chromatin remodeling

Dysregulation of histone modifications and chromatin remodeling forms a central layer of epigenetic control in MB, setting up transcriptional programs that sustain tumor growth and cellular plasticity. In Group 3 MB, aberrant activation of EZH2, the catalytic subunit of PRC2, results in H3K27me3 accumulation, repressing neuronal differentiation genes and thus maintaining tumor cells in a stem-like undifferentiated state characterized by aggressive behavior (Dubuc et al., 2013; Vo et al., 2016).

Imbalance of histone acetylation contributes to transcriptional dysregulation. Hyperactive histone deacetylases (HDACs) promote chromatin compaction and tumor suppressor gene repression, while dysfunction of histone acetyltransferases (HATs), including CBP/p300, impairs activation of differentiation programs (Pei et al., 2016).

Chromatin remodeling is also frequently disrupted. Mutations in SWI/SNF components such as SMARCA4 and ARID1A, particularly in SHH tumors, alter chromatin accessibility and increase reliance on repressive complexes such as PRC1/2 (Johann, 2020).

**FIGURE 1:**
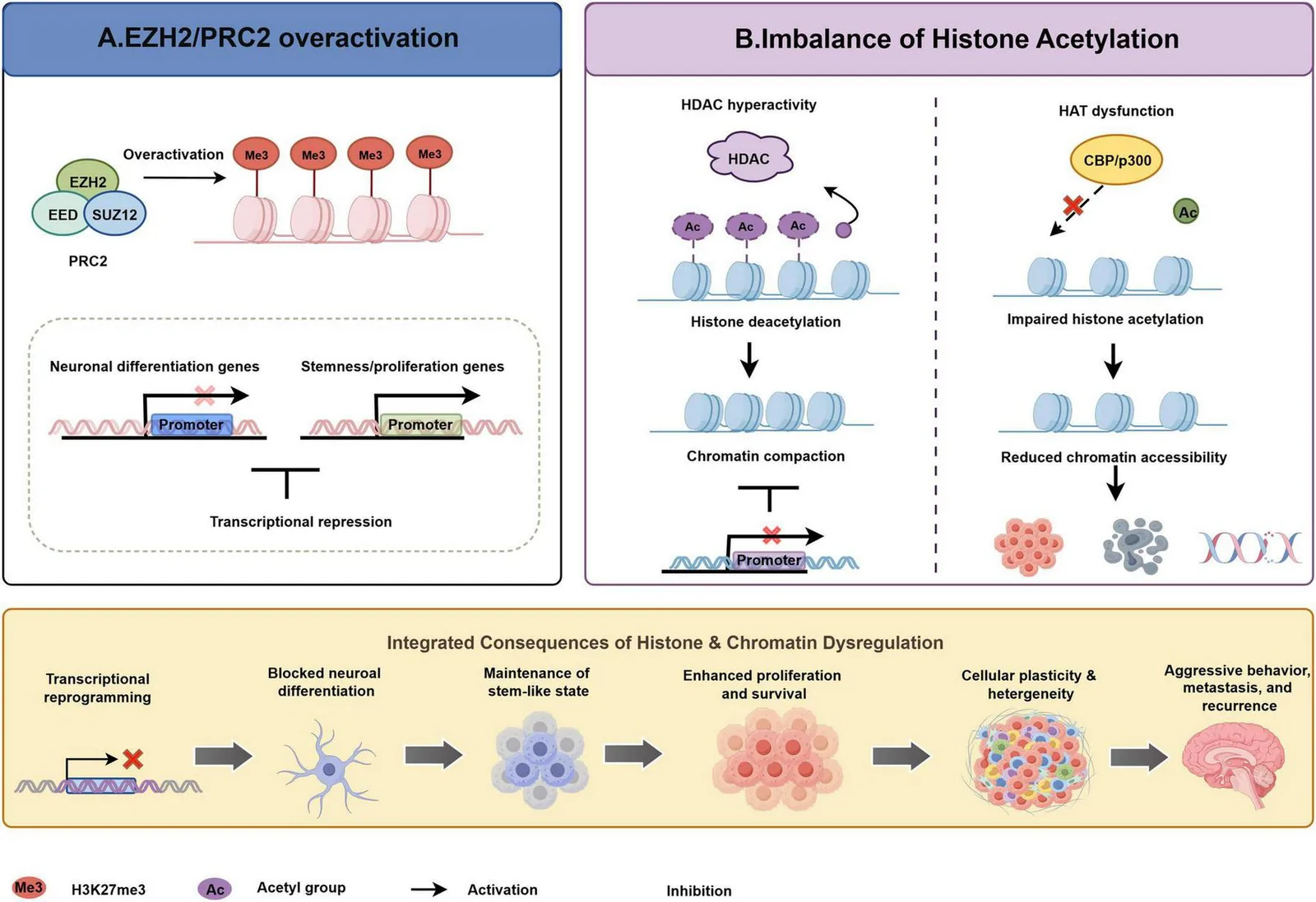
Histone modification dysregulation contributes to medulloblastoma initiation and progression. **(A)** EZH2/PRC2 overactivation in medulloblastoma. Aberrant activation of the polycomb repressive complex 2 (PRC2), composed of enhancer of zeste homolog 2 (EZH2), embryonic ectoderm development (EED), and suppressor of zeste 12 (SUZ12), promotes excessive trimethylation of histone H3 lysine 27 (H3K27me3). The accumulation of H3K27me3 leads to transcriptional repression of neuronal differentiation-associated genes while maintaining the expression programs associated with stemness and proliferation. Consequently, PRC2-mediated epigenetic silencing blocks normal neuronal differentiation and sustains tumor growth. **(B)** Imbalance of histone acetylation in medulloblastoma. Histone acetylation homeostasis is disrupted through either histone deacetylase (HDAC) hyperactivity or dysfunction of histone acetyltransferases (HATs), including CREB-binding protein (CBP) and p300. Excessive histone deacetylation and impaired histone acetylation reduce chromatin accessibility, resulting in transcriptional repression of tumor-suppressive and differentiation-related genes. These alterations contribute to chromatin compaction and epigenetic reprogramming that favors tumor cell survival and expansion. Collectively, dysregulation of histone methylation and acetylation drives widespread transcriptional reprogramming in medulloblastoma, leading to impaired neuronal differentiation, maintenance of stem-like cellular states, enhanced proliferation and survival, increased cellular plasticity and intratumoral heterogeneity, and ultimately aggressive tumor behavior characterized by recurrence and metastasis. This figure was drawn by Figdraw (ID: AWOIS22aac).

All these changes thus confer epigenetic dependencies to tumors that favor targeting EZH2 and HDAC pathways (Figure 1).

### 2.3 Non-coding RNA-mediated regulation

Non-coding RNA (ncRNA) networks represent an additional layer of epigenetic regulation in MB, integrating transcriptional, post-transcriptional, and chromatin-associated mechanisms. Both microRNAs (miRNAs) and long non-coding RNAs (lncRNAs) contribute to tumor initiation, progression, and therapeutic response.

Oncogenic miRNA clusters, such as miR-17∼92, are frequently amplified in Group 3 and Group 4 tumors and thus promote proliferation and invasion by downregulating tumor suppressor pathways, such as PTEN signaling (Murphy et al., 2013; Northcott et al., 2009). Conversely, tumor-suppressive miRNAs are often epigenetically silenced. For example, miR-1253 is downregulated through promoter hypermethylation, and its restoration inhibits tumor growth by targeting CDK6 and CD276, thereby highlighting the interplay between DNA methylation and miRNA regulation (Jackson and Packer, 2023; Northcott et al., 2012).

lncRNAs further modulate chromatin state and gene expression by recruiting chromatin modifiers or acting as competing endogenous RNAs. A few shows subgroup-specific expression linked to metabolic pathways or MYC-driven programs (Li et al., 2009).

In this respect, ncRNA networks further elaborate the complexity of MB epigenetics, thereby offering good prospects for biomarkers and therapeutic targets (Table 1).

**TABLE 1:** Key epigenetic alterations, functional roles, and clinical implications in medulloblastoma.

Epigenetic mechanism	Key regulators/markers	Molecular subgroup (s)	Functional impact	Clinical application	References
DNA methylation	*RASSF1A, HIC1, SPINT2*	SHH, Group 3/4	Gene silencing, genomic instability	Subgroup classification, prognosis	Frühwald et al., 2001; Lusher et al., 2002; Rood et al., 2002
DNA hypomethylation	*MYC*, retrotransposons	Group 3/4	Oncogene activation	Tumor progression marker	Cho et al., 2011; Northcott et al., 2017
Histone methylation	EZH2 (PRC2), H3K27me3	Group3	Stemness maintenance, repression	Target for EZH2 inhibitors	Dubuc et al., 2013; Vo et al., 2016
Histone acetylation	HDACs, CBP/p300	All subgroups	Chromatin compaction, transcription control	HDAC inhibitors	Pei et al., 2016
Chromatin remodeling	SMARCA4, ARID1A (SWI/SNF)	SHH	Chromatin accessibility, differentiation block	Epigenetic vulnerability	Johann, 2020
miRNAs	miR-17∼92, miR-1253	Group 3/4	Cell cycle, apoptosis regulation	Therapeutic targets	Johann, 2020; Murphy et al., 2013; Northcott et al., 2009, 2012
IncRNAs	Inc-HLX-2-7, OTX2-AS1	Multiple	Metabolism, transcription regulation	Biomarkers, drug sensitivity	Jackson and Packer, 2023

### 2.4 Emerging single-cell and multi-omic technologies reveal the dynamic epigenetic landscape of medulloblastoma

Recent advances in single-cell and multi-omic technologies have fundamentally transformed our understanding of medulloblastoma epigenetics (Sheng et al., 2024). While earlier studies primarily relied on bulk genomic and epigenomic profiling, these approaches inevitably masked the extensive cellular heterogeneity present within individual tumors. The emergence of single-cell transcriptomics (scRNA-seq), single-cell chromatin accessibility profiling (scATAC-seq), single-cell DNA methylation analyses, and integrated multi-omic platforms now enables the characterization of epigenetic states at unprecedented resolution, providing new insights into tumor evolution, developmental hierarchies, and therapeutic resistance (Kim and Takahashi, 2025).

Single-cell sequencing studies have revealed that medulloblastoma is composed of multiple transcriptionally and epigenetically distinct cellular populations that coexist within the same tumor. Rather than representing homogeneous molecular subgroups, individual tumors contain diverse cell states spanning stem-like, progenitor-like, proliferative, and differentiated phenotypes (Hovestadt et al., 2019). These cellular states are highly dynamic and are governed by epigenetic regulatory networks that control lineage commitment and developmental plasticity. Such findings are particularly evident in SHH medulloblastoma, where tumor cells closely resemble different stages of granule neuron progenitor differentiation, whereas Group 3 and Group 4 tumors exhibit broader developmental trajectories associated with neuronal and glutamatergic lineage programs (Kiang et al., 2025; Qin et al., 2021). These observations suggest that intratumoral heterogeneity arises not only from genetic alterations but also from dynamic epigenetic regulation that enables cellular state transitions.

Complementing transcriptional profiling, single-cell chromatin accessibility analyses have further demonstrated that subgroup-specific transcriptional programs are orchestrated by distinct cis-regulatory elements and transcription factor networks (Okonechnikov et al., 2025). Integrative analyses combining scRNA-seq with scATAC-seq have identified key regulatory circuits involving MYC, OTX2, ATOH1, NEUROD1, and other lineage-associated transcription factors, providing mechanistic links between chromatin accessibility and subgroup identity (Veo et al., 2025). Importantly, these studies have shown that enhancer activation and chromatin remodeling frequently precede detectable transcriptional changes, suggesting that epigenetic alterations may represent early drivers of tumor progression and phenotypic diversification.

The integration of multiple omic layers has further expanded our understanding of medulloblastoma biology. Recent studies combining genomic, transcriptomic, epigenomic, proteomic, and metabolomic datasets have uncovered coordinated regulatory networks that cannot be resolved using single-platform analyses alone (Okonechnikov et al., 2025). Multi-omic approaches have identified previously unrecognized molecular subtypes, clarified developmental origins, and revealed interactions between epigenetic regulation, metabolic reprogramming, and oncogenic signaling pathways (Veo et al., 2025). These integrated datasets also facilitate the identification of robust biomarkers with improved diagnostic and prognostic performance while providing candidate therapeutic targets for precision medicine (Sheng et al., 2024).

Emerging spatial transcriptomic and three-dimensional epigenomic technologies are adding another layer of complexity by preserving the spatial organization of tumor cells and their microenvironment. Spatially resolved analyses have begun to reveal region-specific epigenetic programs associated with proliferative niches, neuronal differentiation, hypoxic adaptation, and immune cell infiltration (Chien et al., 2024; Li et al., 2025). Similarly, chromosome conformation mapping techniques, including Hi-C and related approaches, have demonstrated that higher-order chromatin architecture contributes to enhancer-promoter interactions and long-range transcriptional regulation in medulloblastoma. These findings suggest that alterations in three-dimensional genome organization may represent an additional epigenetic mechanism driving subgroup-specific gene expression (Sheng et al., 2024).

Collectively, these emerging technologies are shifting the field from descriptive epigenetic profiling toward a multidimensional understanding of regulatory networks operating across individual cells, tissues, and developmental time. Integrating single-cell sequencing, spatial epigenomics, and multi-omic analyses is expected to refine molecular classification, improve biomarker discovery, identify mechanisms of therapeutic resistance, and accelerate the development of precision epigenetic therapies (Kiang et al., 2025; Sheng et al., 2024). As these technologies continue to mature, they will provide an increasingly comprehensive framework for translating epigenetic discoveries into clinically actionable strategies for patients with medulloblastoma.

## 3 Epigenetic heterogeneity, tumor microenvironment, and immune regulation

Beyond tumor initiation, epigenetic alterations shape intratumoral heterogeneity and tumor microenvironment interactions in medulloblastoma. Single-cell and multi-omics studies reveal dynamic, context-dependent regulation driving cellular plasticity, immune modulation, and spatial organization, linking tumor-intrinsic properties with environmental cues and influencing disease progression and therapeutic response (Hovestadt et al., 2019; Vladoiu et al., 2019).

### 3.1 Single-cell epigenetic heterogeneity and cellular plasticity

Recent single-cell transcriptomic and epigenomic profiling revealed high intratumoral heterogeneity in MB, especially concerning epigenetic regulation. In fact, SHH tumors exhibit distinct expression patterns of epigenetic regulators, segregating different populations of malignant cells with subgroup-specific signatures (Hovestadt et al., 2019; Riemondy et al., 2022).

At the single-cell level, epigenetic heterogeneity correlates with functional cell states. Stem-like and proliferative tumor cells exhibit increased epigenetic activity, dysregulated RNA processing, aberrant DNA recombination, and hyperproliferation, which are poor prognostic features (Hovestadt et al., 2019). Notably, MB cells can transit dynamically among multiple transcriptional states, including progenitor-like, cycling, and differentiated phenotypes, underscoring their high cellular plasticity (Garancher et al., 2018; Hovestadt et al., 2019).

Among such plasticity features is the “high cellular plasticity” (HCP) state, associated with altered chromatin accessibility, increased proliferation, and the risk of tumor recurrence. The dynamic state transitions allow tumor cells to evade therapies targeting specific cellular phenotypes (Smith et al., 2022).

Single-cell chromatin accessibility analysis further characterized subgroup-specific regulatory elements and transcription factor networks that drive these transitions, thus mechanistically demonstrating how epigenetic heterogeneity drives oncogenic transcriptional programs (Lin et al., 2016). Moreover, epigenetic heterogeneity interacts with genetic alterations, such as *MYC/MYCN* amplification, to further tumor evolution and resistance to therapy (Vo et al., 2016).

This places epigenetic heterogeneity at the center as a determinant of tumor plasticity, progression, and clinical aggressiveness, which calls for therapeutic strategies directed against dynamic cell states rather than static molecular features.

### 3.2 Epigenetic regulation of the tumor immune microenvironment

Epigenetic mechanisms play a critical role in shaping the tumor immune microenvironment (TIME), influencing immune cell recruitment and immune evasion. For instance, the histone demethylase KDM6A (UTX) promotes expression of Th1-associated chemokines, facilitating recruitment of CD8+T cells and contributing to an immunologically “hot” tumor phenotype (Pan et al., 2018). Emerging evidence from multiple solid tumors indicates that epigenetic mechanisms influence the tumor immune microenvironment. Although similar regulatory mechanisms are increasingly suspected in medulloblastoma, direct evidence remains limited and warrants further investigation.

Conversely, epigenetic silencing mechanisms, mainly DNA hypermethylation of tumor-associated antigens and genes involved in antigen presentation, reduce tumor immunogenicity, leading to immune escape and formation of “cold” tumors (Topper et al., 2017). While such direct evidence is still sparse for MB, well-established mechanisms in other tumor types support them.

Epigenetic regulation also mediates the spatial organization of tumor cells in the microenvironment. MB tumors present a spatially organized niche of stem-like, proliferative, and more differentiated cells that occupy different topographies (Chien et al., 2024). This spatial pattern results from an interaction between microenvironmental cues and epigenetic programs regulating stemness and differentiation (Lin et al., 2016; Vo et al., 2016).

Recent work has thus uncovered subgroup-specific regulatory circuits of transcription factors and signaling pathways that underlie tumor biology and immune interaction. Epigenetic pathways represent a dual therapeutic opportunity: tumor growth suppression and TIME reprogramming.

This will convert “cold” tumors into “hot” tumors, making them susceptible to immunotherapies like CAR T-cell therapy and antibody therapies (Dunn et al., 2004). Therefore, epigenetic regulation is a significant interface between tumor-intrinsic programs and the immune microenvironment, providing a strong rationale for combined therapeutic strategies.

These findings thus place epigenetic plasticity as one of the leading factors in tumor heterogeneity and microenvironment interaction, underlining the central place of epigenetic plasticity in disease progression and therapeutic resistance.

## 4 Diagnostic and prognostic biomarkers based on epigenetic mechanisms

Advances in epigenetic research into MB have led to clinically relevant biomarkers in diagnostic, prognostic, and therapeutic stratification fields. DNA methylation profiling underlies molecular classification, while histone modifications, chromatin regulators, and non-coding RNAs provide complementary information to support risk stratification and disease monitoring for precision medicine (Capper et al., 2018; Northcott et al., 2019).

### 4.1 DNA methylation profiles as tools for molecular subtyping and prognosis

Non-coding RNA (ncRNA) networks represent an additional layer of epigenetic regulation in MB, integrating transcriptional, post-transcriptional, and chromatin-associated mechanisms. Both microRNAs (miRNAs) and lncRNAs contribute to tumor initiation, progression, and therapeutic response (Hovestadt et al., 2020).

Oncogenic miRNA clusters, such as miR-17∼92, are frequently amplified in Group 3 and Group 4 tumors, promoting proliferation and invasion by repressing tumor suppressor pathways, including PTEN signaling (Murphy et al., 2013; Northcott et al., 2009). On the other hand, tumor-suppressive miRNAs are epigenetically silenced. For example, miR-1253 is downregulated due to promoter hypermethylation. Its restoration inhibits tumor growth by targeting CDK6 and CD276, thus demonstrating functional cooperation between DNA methylation and miRNA regulation (Ferretti et al., 2008).

lncRNAs also regulate chromatin states and gene expression by recruiting chromatin modifiers or acting as competing endogenous RNAs (Laneve et al., 2019; Nejadi Orang and Abdoli Shadbad, 2024). Some are expressed in a subgroup-specific manner and are related to metabolic pathways or MYC-driven programs (Laneve and Caffarelli, 2020; Nejadi Orang and Abdoli Shadbad, 2024). Overall, these ncRNA regulatory networks add another level of complexity to the epigenetic landscape of MB and represent a promising source of biomarkers and therapeutic targets.

### 4.2 Histone modifications and chromatin regulators as biomarkers

Histone modifications and chromatin regulators represent additional epigenetic layers that may also have diagnostic and prognostic value in MB. Components of chromatin-modifying complexes, particularly Polycomb Repressive Complex 2 (PRC2), are associated with tumor origin, differentiation status, and aggressiveness, although their predictive value requires further validation (Dubuc et al., 2013). Interactions between chromatin regulation and non-coding RNAs are increasingly recognized; for example, the lncRNA OTX2-AS1 promotes tumor growth and may predict response to BCL-2 inhibition (Badodi et al., 2017). Genome-wide analyses also reveal sex-specific epigenetic patterns, especially in SHH MB, with differential methylation correlating with survival outcomes (Sharma et al., 2019).

Beyond genome-wide methylation profiling, immunohistochemical markers such as β-catenin, YAP1, GAB1, and LEF1 provide practical surrogates for molecular subtyping. Collectively, these features expand epigenetic biomarkers for classification, prognosis, and therapeutic stratification in MB (Ellison et al., 2011).

### 4.3 Clinical application and translational perspectives of epigenetic biomarkers

Epigenetic research has quickly translated biomarkers into clinical practice in MB. DNA methylation profiling is currently used for molecular classification and risk stratification, while ctDNA methylation in the cerebrospinal fluid is emerging as a minimally invasive tool for real-time monitoring and detection of recurrence (Greuter et al., 2022). Epigenetic biomarkers integrated with clinical and genetic information further improve the prognostic model and contribute to the personalization of treatment (Capper et al., 2018). Moreover, epigenetic signatures may serve as markers for responses to targeted treatments with HDAC and EZH2 inhibitors, as well as immunotherapy. Epigenetic biomarkers provide the dual diagnostic and therapeutic value of precision medicine for MB (Pei et al., 2016).

## 5 Epigenetic therapeutic strategies

Recent advances in cancer epigenetics have accelerated the development of epigenetic-based therapeutic strategies for medulloblastoma. Among these, DNA methyltransferase (DNMT) inhibitors, including decitabine and azacitidine, have demonstrated the ability to reverse aberrant DNA methylation, restore the expression of tumor suppressor genes, and enhance the sensitivity of medulloblastoma cells to conventional chemotherapy in preclinical studies (Gorini et al., 2023; Skouras et al., 2023). Histone deacetylase (HDAC) inhibitors, such as panobinostat, vorinostat, and the dual PI3K/HDAC inhibitor fimepinostat (CUDC-907), exhibit potent antitumor activity by inducing cell-cycle arrest, apoptosis, and differentiation while suppressing oncogenic signaling pathways. Notably, fimepinostat is currently being evaluated in children with recurrent medulloblastoma and other pediatric brain tumors in the PNOC016 clinical trial (NCT03893487) (Gorini et al., 2023; Liu et al., 2026). In addition, inhibitors targeting histone methylation regulators have attracted increasing attention. The EZH2 inhibitor tazemetostat, which has received FDA approval for several malignancies, is currently under clinical investigation in pediatric patients with recurrent medulloblastoma (NCT03155620), reflecting the therapeutic potential of targeting PRC2-mediated epigenetic dysregulation (Gold and Shilatifard, 2024; Liu et al., 2026). Bromodomain and extraterminal domain (BET) inhibitors, including JQ1 and OTX015 (birabresib), effectively suppress MYC/MYCN-dependent transcriptional programs and inhibit tumor growth in MYC-driven Group 3 and SHH medulloblastoma models (Bandopadhayay et al., 2014; Kling et al., 2022). Emerging evidence also suggests that LSD1 inhibitors, such as SP2509 and ORY-1001, impair tumor cell self-renewal and promote neuronal differentiation in preclinical medulloblastoma models (Bibbò et al., 2024). Although most epigenetic agents remain at the preclinical or early-phase clinical stage, accumulating evidence indicates that rational combinations of epigenetic drugs with chemotherapy, radiotherapy, or immunotherapy may enhance therapeutic efficacy while overcoming treatment resistance in medulloblastoma (Liu et al., 2026).

## 6 Discussion

Epigenetic dysregulation has increasingly been viewed as a hallmark of MB biology, not just a downstream consequence of genetic changes but a driving force behind tumor initiation, progression, and resistance to treatment (Hovestadt et al., 2020; Northcott et al., 2019). This paradigm applies especially well to Group 3 and 4 tumors, which have a relatively low burden of recurrent genetic mutations and are highly dependent on epigenetic mechanisms (Northcott et al., 2017).

On the mechanistic level, aberrant DNA methylation, histone modifications, chromatin remodeling, and ncRNA-mediated regulation coordinate to set oncogenic transcriptional programs while disrupting normal developmental trajectories (Dubuc et al., 2013; Northcott et al., 2019). They act as highly interconnected regulatory networks that reinforce tumor cell identity yet allow for adaptive responses to environmental and therapeutic pressures. In addition, epigenetic regulation has been increasingly implicated in coupling metabolic reprogramming and signaling pathways into dynamic feedback loops that support tumor growth and phenotypic plasticity (Vander Heiden and DeBerardinis, 2017).

A recent major conceptual breakthrough was recognizing that epigenetic dysregulation forms the basis of intratumoral heterogeneity and cellular plasticity. Single-cell analyses have shown that MB cells dynamically epigenetically remodel and switch between different functional states, including stem-like and differentiated phenotypes (Hovestadt et al., 2019; Vladoiu et al., 2019). Such plasticity is a fundamental barrier to effective treatment, as it allows tumor cells to escape therapies targeting particular cellular states. At the same time, epigenetic mechanisms shape the tumor immune microenvironment by modulating immune cell recruitment and antigen presentation, thereby participating in immune evasion (Vladoiu et al., 2019).

We propose a conceptual model in which epigenetic regulation represents a critical integrative hub connecting tumor-intrinsic transcriptional programs, cellular state transitions, and microenvironmental interactions. In this respect, epigenetic plasticity drives tumor heterogeneity and adaptive resistance to therapy, representing a crucial determinant of clinical outcomes.

From a translational perspective, DNA methylation profiling has become the gold standard for MB classification and risk stratification, while emerging approaches such as cerebrospinal fluid-derived ctDNA methylation analysis enable non-invasive disease monitoring (Capper et al., 2018). Additional epigenetic layers, including histone modifications and ncRNA networks, further enhance diagnostic and prognostic precision (Northcott et al., 2009). However, these advances in epigenetic-targeted therapies are hampered by subgroup-specific heterogeneity and dynamic changes in epigenetic states, warranting rational combination strategies, particularly with immunotherapy (Topper et al., 2017).

In future research, multi-omics, single-cell, and spatial profiling will be integrated to elaborate on epigenetic dynamics in MB (Hovestadt et al., 2019; Lin et al., 2016). Therefore, predictive biomarkers and combination therapies based on epigenetic plasticity will have to be developed to translate these insights into lasting clinical benefits.

## 7 Conclusion

Epigenetic dysregulation is one of the central determinants of MB biology regarding tumor initiation, progression, cellular heterogeneity, and therapeutic response (Hovestadt et al., 2020; Northcott et al., 2019). DNA methylation, histone modifications, chromatin remodeling, and non-coding RNA regulation collectively establish transcriptional programs that sustain tumor cell identity and plasticity, while also influencing the tumor immune microenvironment (Northcott et al., 2019; Pan et al., 2018).

These advances have translated into clinically relevant applications, particularly in biomarker development. DNA methylation profiling has redefined molecular classification and risk stratification, and liquid biopsy approaches offer new opportunities for non-invasive disease monitoring. Epigenetic alterations are also being explored as predictive markers and therapeutic targets.

Clinical application is hampered by epigenetic heterogeneity and dynamic state changes. Integrative multi-omics approaches and rational combination strategies will be needed to translate these insights into improved outcomes for MB patients (Hovestadt et al., 2019; Pan et al., 2018).

Therefore, placing epigenetic regulation at the center of a unifying integrative hub places medulloblastoma biology within a strategic framework for the development of biomarker-driven combination therapeutic approaches.

Future studies should move beyond describing individual epigenetic alterations toward integrating single-cell, spatial, and multi-omic datasets to define dynamic regulatory networks underlying tumor evolution and treatment resistance. Such integrative approaches will facilitate the identification of clinically actionable biomarkers and novel therapeutic targets while enabling patient stratification and personalized epigenetic interventions. We anticipate that combining high-resolution epigenomic profiling with functional validation and clinical translation will represent the next stage of precision medicine for medulloblastoma.
